# Posterior Interosseous Nerve Syndrome from Thermal Injury

**DOI:** 10.1155/2014/891393

**Published:** 2014-03-06

**Authors:** Vijay A. Singh, Rami E. Michael, Duy-Bao P. Dinh, Scott Bloom, Michael Cooper

**Affiliations:** ^1^Department of Surgery, Staten Island University Hospital, Staten Island, NY, USA; ^2^Division of Burns, Department of Surgery, Staten Island University Hospital, Staten Island, NY, USA

## Abstract

*Background*. Due to anatomical proximity to bone, the radial nerve is the most frequently injured major nerve of the upper extremity, frequently secondary to fractures (Li et al. (2013)). We describe an incidence when a branch of the radial nerve is injured as a result of a thermal injury. *Observation*. Radial nerve injury can occur anywhere along the anatomical course with varied etiologies, but commonly related to trauma. The most frequent site is in the proximal forearm involving the posterior interosseous branch. However, problems can occur at the junction of the middle and proximal thirds of the humerus and wrist radially. When the radial nerve is injured by a burn, a new rehabilitation dynamic arises. Not only does one agonize about the return of nerve function but also fret about the skin grafts that replaced the devitalized tissue housing that compartment. *Discussion*. Although posterior interosseous nerve syndrome has been described in the context of many different etiologies, it has not previously been discussed in relation to burn injuries. In this case, not only did the patient's rehabilitation involve aggressive therapy for return of sensation and function of the arm, but also prevention of contracture normally seen in replacement of full thickness burns.

## 1. Introduction

Due to its anatomy, the radial nerve is the most frequently injured major nerve of the upper extremity [1]. Proximally, the nerve is closely related to the shaft of the humerus, while at the elbow, it divides into superficial and posterior interosseous branches, the latter of which is closely related to the neck of the radius [1, 2]. Given the close proximity of the nerve to the bone along its course, it is frequently damaged in the context of closed fractures to the upper extremity [1, 2]. It is estimated that as many as 12% of humeral shaft fractures are associated with radial nerve paralysis [3].

The posterior interosseous nerve is the deep motor branch of the radial nerve supplying the majority of the forearm and hand extensors (including the extensor carpi radialis brevis, supinator, extensor digitorum communis, extensor digiti quinti, extensor carpi ulnaris, abductor pollicis longus and brevis, and extensor indicis proprius) [1].

Damage to this branch of the radial nerve results in the posterior interosseous nerve syndrome, a pattern seen when the muscles supplied by this nerve do not function [4]. Classically, the patient is unable to extend the thumb and fingers of the involved extremity at their metacarpophalangeal joints [5]. These patients may be able to dorsiflex at the wrist but only in a dorsoradial direction [6].

The most common etiology for radial nerve compression is by way of traumatic injury; other causes include tumors, inflammation, and anatomic compression [7]. In addition to closed fractures secondary to direct trauma, compressive injuries to the upper extremity, radial nerve compression may also result in damage to the radial nerve along its course [8]. Here we present a rare case of posterior interosseous nerve damage secondary to a compressive burn injury.

## 2. Case Presentation

A 34-year-old male patient who was previously healthy suffered 20% total body surface area mechanical burns and crush injuries to his bilateral upper extremities and right lower extremity, after being caught in a cucumber stripping machine. He was initially treated for his traumatic injuries at the regional trauma center and was noted to have rhabdomyolysis associated with traumatic muscle injury. The patient was fluid resuscitated, ruled out for any additional traumatic injuries and subsequently transferred to a burn center for care of his thermal injuries.

**Figure 1:**
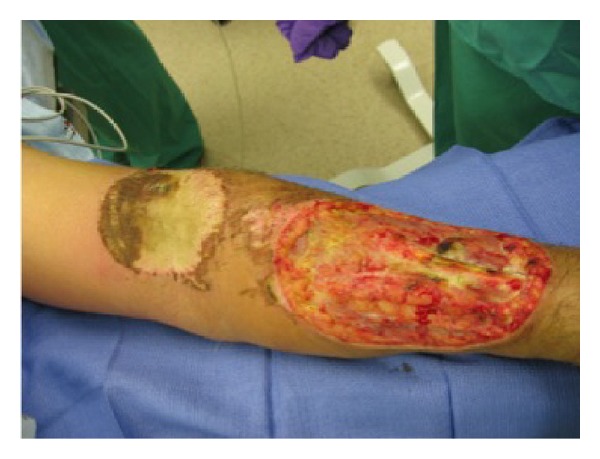
Left upper extremity thermal injury.

Worthy of note is that on examination the patient was unable to extend his left hand and fingers. In addition, he was found to have necrosis and cellulitis involving the burned areas of his left upper extremity, which appeared to be rapidly progressing. He was therefore taken to the operating room for debridement of the wound (Figure 1). At that time, both upper extremity wounds as well as the wound to his right lower extremity were excised of devitalized tissue. The necrotic area was excised down to the fascia, and necrotic muscle tissue was removed wherever possible.

Postoperatively the patient's myoglobin and creatinine kinase normalized with intravenous hydration. He was started on empiric antibiotic therapy, and his intraoperative wound cultures eventually grew several pansensitive organisms including *Pseudomonas aeruginosa*, *Aeromonas hydrophila*, *Enterococcus faecalis*, *Enterobacter cloacae*, and *Proteus vulgaris*, all of which were amenable to treatment with oral gatifloxacin, for which he was eventually switched too.

The patient returned to the operating room three days after admission for possible further debridement of the wounds as well as exploration of his left radial nerve, at which time additional necrotic areas were noted in the left forearm wound extending proximally. These areas were excised completely, taking care to preserve the neurovascular structures.

The left arm wound was extended proximally to expose the length of the biceps and posteriorly to include the elbow. Similarly, on the right arm, the wound was extended and excised. After all necrotic tissue was debrided, each of his wounds was covered with allograft (donor skin) meshed to a ratio of 2 : 1 and dressed. The left hand was then splinted in neutral position. The left elbow was also splinted. On hospital day five, these dressings were removed revealing healthy adherent allograft to the previous wounded areas. Given the patent's left upper extremity palsy, he was referred for electrodiagnostic testing to evaluate for the presence of a radial neuropathy. He was found to have an absent left radial nerve sensory response. This exam was unable, however, to elucidate the etiology of the conduction loss, but given the manner in which this injury was sustained and his presentation with rhabdomyolysis and necrotic muscle, it is likely that the patient had sustained a crush injury in addition to his burns.

On hospital day twelve, the patient was discharged with plans to return to the operating room for exploration of the left upper extremity in order to assess nerve damage as well as to place autografts on previously allografted areas.

**Figure 2:**
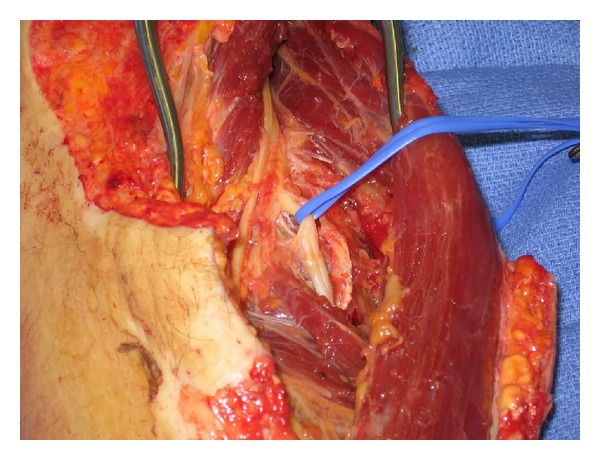
Radial nerve isolated in center of picture.

He was readmitted approximately four days after discharge for this planed exploration and autografting. The left forearm and radial nerve were explored at this time revealing no evidence of direct injury to the nerve (Figure 2). The left forearm was initially explored between the brachioradialis and the extensor carpi radialis longus, where the superficial radial nerve was emerging between the brachioradialis and the extensor carpi radialis longus. The muscles were split to see the superficial radial nerve overlying the supinator and the posterior interosseous nerve extending beneath the supinator itself.

The recurrent radial artery and its accompanying veins were divided. The posterior interosseous nerve was then traced in its course and was found to be crushed with abundant scar tissue around it. The scar was carefully divided, and the nerve was followed until it appeared healthy and branched into the extensor muscles of the forearm and thumb.

During the course of his outpatient followup, the patient's left antecubital fossa demonstrated a well granulating wound; however, a contracture over this area limited the range of motion about his elbow to 25 degrees short of full extension. He was then readmitted for release of the burn scar contracture to his left elbow. The left elbow contracture was released via a horizontal incision across the antecubital fossa until the range of motion about the elbow was limited to only approximately two degrees short of full extension. Approximately 200 sq cm of 1 : 1 meshed split thickness skin harvested from his left thigh was placed over the left antecubital fossa wound and anchored into place with skin staples.

The patient's postoperative course was uneventful. He will be followed in our clinic on a routine basis for further evaluation of his autografts and left radial nerve palsy. Although the prognosis following posterior interosseous nerve decompression is reported to be unpredictable, we are hopeful that this patient's function will improve. At last report, the patient has had some improvement in function of his left upper extremity.

## 3. Discussion

Although posterior interosseous nerve syndrome has been described in the context of soft-tissue tumors, synovial proliferation in rheumatoid disease, fractures, and dislocations of the head of the radius, osteomyelitis of the proximal radius, and Volkmann's ischemia of the forearm, it has not been described in the context of burn/crush injuries. In general, crush injuries present a unique challenge due to combined injuries of the skin, the underlying soft tissues and muscle, as well as the possibility of bony fractures and tendon damage. Future research is necessary to further classify neuropathy after burn injuries, not only in terms of the nature of the direct mechanism of injury (thermal injury, compression, and vascular occlusion of the vasa nervorum) but also in terms of long term prognosis and rates of recovery from various injury classifications. Animal experiments have revealed that crush duration is an important factor in nerve damage and recovery of function at low crushing levels, but at higher crush levels the degree of mechanical insult correlates with recovery of function. Posterior interosseous nerve syndrome may be associated with burn/crush injuries in the forearm.

Aggressive medical and surgical management is essential in the early management of patients who present with crush injuries. This case also highlights the fact that surgical debridement of all compromised tissues is necessary for a favorable outcome in this setting.
